# The Individual Profile of Pathology as a New Model for Filling Knowledge Gaps in Health Policies for Chronicity

**DOI:** 10.3389/fmed.2019.00130

**Published:** 2019-06-13

**Authors:** Michela Franchini, Stefania Pieroni, Arianna Cutilli, Michelangelo Caiolfa, Simone Naldoni, Sabrina Molinaro

**Affiliations:** ^1^Institute of Clinical Physiology, National Research Council, Pisa, Italy; ^2^Federsanità-Anci Toscana, Firenze, Italy

**Keywords:** long term conditions, co-morbidity, complex needs, general population, algorithm, segmentation, health policy

## Abstract

Chronicity is the real challenge for public healthcare systems especially in relation to multi-morbidity. The growing demand for multidisciplinary care could be addressed by implementing integrated programs in the primary care field and facilitating other specific care only as necessary. Some models of long-term management have been suggested since the 2000s. The objective here is to propose the Individual Profile of Pathology (IPP) model as the preliminary step for identifying groups of population which shares health and social needs and for optimizing the management of chronicity, referring to the Kaiser Permanente Pyramid paradigm. The IPP model is able to inform a data feedback system for improving performances at the patient's individual level and for addressing and evaluating health policies. The stratification of needs comes out of the IPP algorithm. It works on patient information databases based on the logic of disease as a process that evolves over time and interacts with many factors unique to that patient. Individual patients' data used in this work refers to 138,859 subjects from a large area in Italy and concerns hospitalization, outpatient drug prescriptions, access to the emergency room and outpatient prescriptions for visits, laboratory/imaging tests, and medications. The IPP model allows to identify for each subject a complexity level, taking into account the weight of groups of pathologies, both in terms of absorption of resources and the level of severity. Costs and healthcare performances have been analyzed taking into account the complexity levels. The IPP model can be an efficient methodology for (a) improving performances at the patient's individual level (b) allowing standardized comparison among different geographical areas (c) supporting large population-focused surveillance programs and (d) providing knowledge to identify and fill the gaps in public health policies. Currently, the IPP algorithm is limited by data availability, restricted to the administrative databases processing, but the theoretical model is able to include more data dimensions providing the potential to identify homogeneous groups of subjects with a higher level of precision.

## Introduction

Most OECD countries currently allocate between about 1 and 1.5% of their GDP to long-term conditions (LTC) and LTC expenditure are expected to fall in the range of 2.2–2.9% of GPD in 2050 (1) because disability is causing the greatest fraction of the burden of disease as demographics and epidemiology change.

Currently, the disease burden is caused mostly by chronic diseases and injuries and this burden intensifies as people live longer.

The types of illnesses and injuries causing death and disability are also changing. Ischemic heart disease and stroke remained the two greatest causes of death between 1990 and 2010 and projections to 2030 indicate that these ailments will remain leading causes. Conversely, all the other rankings in the top 10 causes of death changed. Diseases such as diabetes, lung cancer, and COPD moved up, and diarrhea, lower respiratory infections, and tuberculosis moved down (2).

Projections to 2030 also indicate that COPD will move up, reflecting in part the projected increases in death and disability from tobacco use (2).

Effective chronic illness interventions generally rely on multidisciplinary care teams (3) and Integrated Care Pathways. These can have positive effects on service quality and efficiency by supporting the timely implementation of clinical interventions and the mobilization of resources around the patient, without incurring in additional increases of length of stay (4).

The growing demand for multidisciplinary care could be addressed by implementing integrated programs in the primary care field and facilitating secondary care, hospitalization, and tertiary care only as necessary (5, 6).

Addressing the complex needs of people suffering from multiple chronic diseases requires a holistic and proactive approach, patient focused, that implies a more efficient use of resources (7).

Some models of long-term care management have been suggested since the 2000s as the Chronic Care Model and the Kaiser Permanente Pyramid (8).

Both models are based on systematic process that identifies groups of population that may benefit from specific clinical intervention.

The Kaiser Permanente Pyramid, in particular, stratifies health and social needs of patients with chronic disease on three groups, depending on the level of management that they should optimally require: (a) self-care approach involving education about health condition/s, strategies to manage changes in own health and about the importance of compliance to drug prescription, (b) disease-specific care management based on evidence based guidelines and supported by multi-disciplinary teams of clinicians and (c) case management regarding patients with multiple long-term conditions whose complexity makes them high users of healthcare and social services.

The Kaiser Permanente Pyramid groups could be better identified using systematic processes of risk stratification for case finding, based on clinical data.

On the basis of different informative purposes, there are several approaches to risk stratification and each has advantages and limits.

Methods to segment and stratify populations generally use different sources of data: (a) quantitative data, such as administrative data and claims-based algorithms, (b) qualitative data including clinical judgment and referral, (c) hybrid approaches that incorporate both qualitative and quantitative data (9, 10).

Currently, hybrid approaches seem to be more efficient than using single source of data (9).

We have developed and indirectly validated, the Individual Profile of Pathology (IPP) algorithm for supplying disease and patient stratification using administrative databases which contain mainly demographics and health/disease history.

The IPPs stratification refers to the concept of disease as a process that evolves over time (11) and is characterized by some markers that could be detected before the disease onset and that could support a proactive approach toward the patient.

The idea is that the combination of the different macro-categories of comorbidities, which the patient belongs to, could drive more efficiently the identification of the diagnostic and therapeutic pathways at the individual level and could improve the integration among different clinicians, care providers and settings. So, the IPP stratification model could support in a more appropriate way the management of chronic disease.

In other words, our aim is to propose the IPP model as the preliminary step for optimizing the management of chronicity, referring to the Kaiser Permanente Pyramid paradigm which links the concept of health need, with the concepts of pathway of care and resources management in chronicity.

Currently, the individual profiles (IPPs) derive from the combination of quantitative and qualitative data extracted from claim-based sources of individual information while the needs' stratification proceeds from the use of appropriate algorithms working on IPPs.

Ideally we should be able to integrate in the IPP model other sources of individual data as (a) socio-economic factors, family status, quality of life, employment type etc. (b) disease analytics information as complexity of diagnosis, susceptibilities, age of onset, impact on life expectancy and quality, potential for prevention, (c) genetic profile as biological markers and (d) environmental exposure/air pollution data sources for effectively implementing personalized medicine solutions.

## Methods

### Study Population and Data Sources

The study population (138,859 subjects) is composed of all residents in two different geographical areas in Italy (*n* = 80,641 Area 1; *n* = 58,218 Area 2) with similar demographic structure.

The subjects were characterized by integrating and harmonizing relevant data derived from their contacts with the Italian National Healthcare Service (NHS) in 2013 and 2014, and collected in specific data flows of standardized information from different data sources (12): (a) an archive of all residents in those areas receiving NHS assistance storing demographic and administrative data, including the GP's identifier of each subject, (b) hospital discharge forms from public and/or private hospitals reporting all diagnoses related to hospitalizations, (c) outpatient drug prescriptions reimbursable by the NHS, (d) outpatient prescriptions for visits, laboratory/imaging tests, and medications, (e) mortality data, (f) access to the emergency room (ER), (g) pathology registers that contain reporting from GPs about all their patients presenting Heart Failure and Diabetes and (h) registers of all patients receiving home assistance.

**Figure 1:**
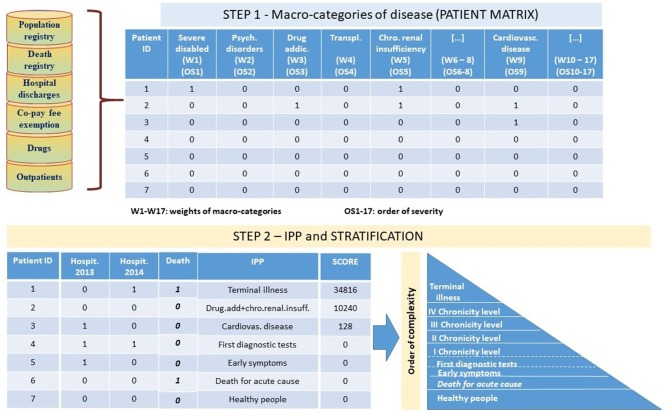
The IPP algorithm.

The IPP algorithm, has been implemented in two-steps (Figure 1). In the first step, a secondary analysis of the integrated information from sources described before and in particular (a) identified as “Population Registry” and “Co-pay fee exemption” in the Figure 1, (b) identified as “Hospital discharges” in the Figure 1, (c) identified as “Drugs” in the Figure 1, (d) identified as “Outpatients” in the Figure 1, (e) identified as “Death registry” in the Figure 1 allowed to identify for each subject all the macro-categories of pathology to which she/he belongs to.

For this purpose, we adopted and upgraded a classification system originally developed by Pavia Local Health Unit in collaboration with Pavia University (13). Through this approach, each subject is placed within pathological macro-categories based on criteria that consider pathology exemption codes, diagnosis and treatments from hospital discharge and outpatients, and consumption of drugs. As an example, a patient is considered neoplastic if she/he received outpatient treatment in the oncology branch and/or she/he took antineoplastic drugs and/or she/he had diagnosis of tumor (code between 140^*^ and 208^*^ considering ICD9CM classification) during hospital stays.

According to the model, having linked all the available information to each subject and having applied classification criteria as described, we created a patient table structure, referred as patient matrix in Figure 1. The patient table contains one row for each subject and 18 separate columns identifying the different macro-categories of disease including “Death.” The columns are filled by dichotomous variables where the value 1 indicates the presence of the disease for the subject and the value 0 indicates the absence of the disease, and are positioned in descending order of severity: severe disabled, psychiatric disorders, drug addictions, transplants, chronic renal insufficiency, HIV positive and AIDS acclaimed, neoplasm, diabetes, cardiovascular diseases, chronic obstructive pulmonary, gastro-enteropathy, neuropathy, autoimmune, endocrine and metabolic diseases, rare diseases, pregnant women, other residual pathologies.

Death is considered separately in the second step of the algorithm (Figure 1).

The created patient table is the input for the step 2 of the algorithm.

In this step the macro-categories of each patient have been combined into his IPP (Figure 1). The combined score of each individual profile is the sum of the weights of the macro-categories included in the profile based on the order of complexity identified by the algorithm—e.g., a patient presenting cardiovascular, neoplasm and gastro-enteropathy co-morbidities has a higher score than a patient presenting cardiovascular, chronic obstructive pulmonary, and gastro-enteropathy.

Not-chronic patients have IPP scores equal to zero. Within this group, patients differ on the basis of hospitalization events (early symptoms/first diagnostic tests) or mortality (death for acute cause).

Chronic patients have IPP scores <0. We have identified four levels of chronicity (I–IV) considering the quartiles of the distribution of scores weighted for the proportion of people who died in each IPP.

Moreover, mortality data have been used to identify those chronic patients with the highest level of complexity within the same IPP: terminal patients are subjects belonging to the four chronic levels and who died during one of the years of observation.

**Figure 2:**
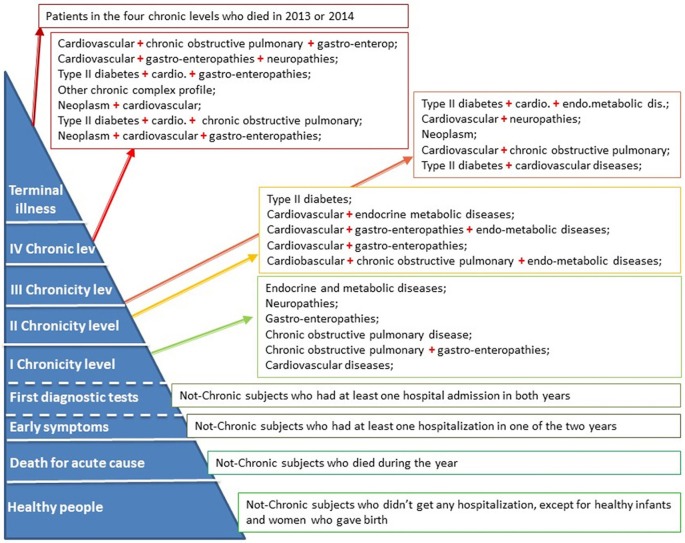
The IPP model.

In particular the IPP model identified the following levels, as shown in Figure 2: (a) “Healthy people” includes the subjects who do not have chronic conditions and who have not undergone any hospitalization (except for healthy newborns and women giving birth); (b) “Death for acute cause” are the subjects who have died in the year without any chronic diseases; (c) “Early symptoms” level refers to the alive not-chronic subjects who have had at least one hospital admission in one of the 2 years; (d) “First diagnostic tests” level includes the not-chronic patients who have had at least one hospital admission in both years; (e) four separate chronic levels characterized by different co-morbidity combinations and (f) the “Terminal illness” level which refers to patients belonging to the four chronic levels and who have died in 2013 or 2014.

The IPP model was used to perform specific analysis on: (a) hospitalization demand, (b) ER access, (c) home and/or community care service and (d) health spending, in order to identify the specificities of each level of complexity, for supporting a better pathway of care and resources management. The IPP model was also used for monitoring the pathology complexity progression over time.

Moreover, we applied the model on data from two different geographical areas with similar demographic structure (Area 1 and Area 2) in order to indirectly validate the model, so implementing a first level of validation.

## Results

### Distribution of Population by Levels of Complexity

In both areas, the results from the IPP algorithm show that chronic patients amount to the 39.4% of the study populations. The 16% of those patients is affected by at least two pathologies belonging to different diagnostic categories.

**Figure 3:**
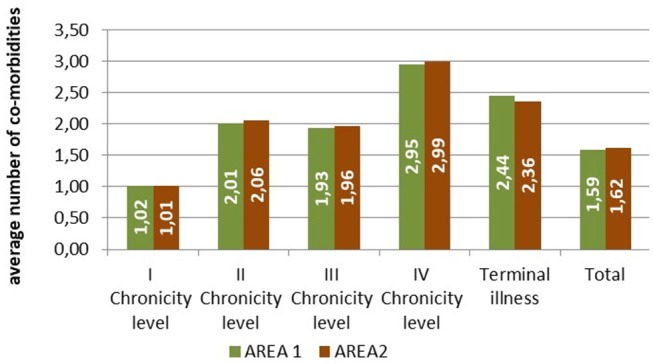
Average number of comorbidities belonging to different diagnostic categories by level of complexity.

The patients belonging to the fourth level of chronicity showed the highest number of comorbidities belonging to different diagnostic categories, followed by the patients in the terminal state (Figure 3).

The first chronic level, affecting the 22.2% of the population (Area1: 21.6%; Area2: 23.0%), includes patients belonging to a single diagnostic category (cardiovascular, endo-metabolic, neurological, gastro-enterological, chronic obstructive pulmonary diseases) or patients affected both by bronchopulmonary and gastroenterological diseases.

The cardiovascular diseases are the main health problems in the second chronic level which includes 5.1% of the population (Area1: 5.1%; Area2: 5.2%). The cardiovascular diseases are frequently associated to another morbidity as chronic obstructive pulmonary, gastroenterological, endometabolic pathologies. The second level also includes patients with type 2 diabetes.

Within the third level of chronicity (5.3% of the population; Area1: 5.1%; Area2: 5.7%), the individual profiles include more than 2 categories of diseases except for neoplasms appearing in an unbound form. Neoplasms combined with other diagnostic categories characterize the fourth chronic level (5.9% of the population; Area1: 5.6%; Area2: 6.5%). The fourth level also refers to patients with other chronic complex profiles which altogether amount to <4% of the population.

The patients in the terminal state (1.1% of the population; Area1: 1.1%; Area2: 1.2%) include those who died in 2013 or 2014 for the worsening of their chronic conditions. Of these patients, the 6% is derived from the first chronic level, the 26% from the second, the 21% from the third and the most (48%) from the highest level of chronicity.

**Figure 4:**
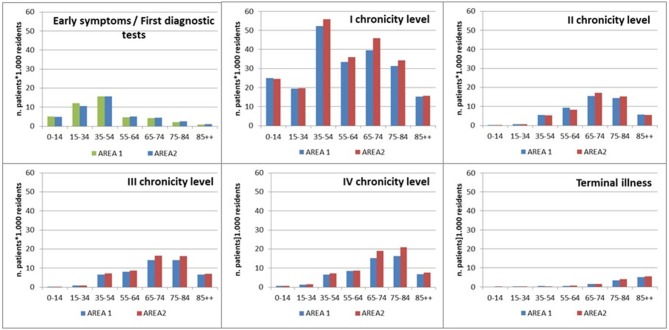
Distribution by levels of complexity and age class.

As shown in Figure 4, the distribution of population by age within each level of complexity showed a common increased trend until 65–74 years of age, from the second to the fourth level. The “Early symptoms/initial investigation” level mainly concerned people younger than 54 years of age, while the first chronicity level showed a bimodal distribution referring to 35–54 and 65–74 years of age. The “Terminal illness” level showed an increasing trend from the youngest to the oldest age class.

The results from the application of the IPP model in Area 1 and Area 2 were very similar.

### Distribution of Hospitalization by Levels of Complexity

The study population in 2013 showed overall hospitalization rate which differ among Area 1 and Area2. Area 2, in particular, had higher hospitalization rates for both gender (women 102.1 vs. 90.7 ^*^1.000; men 108.3 vs. 88.0 ^*^1.000), even though the distribution among the complexity levels is quite similar.

The patients in terminal state were more frequently hospitalized than others, in particular among men.

**Figure 5:**
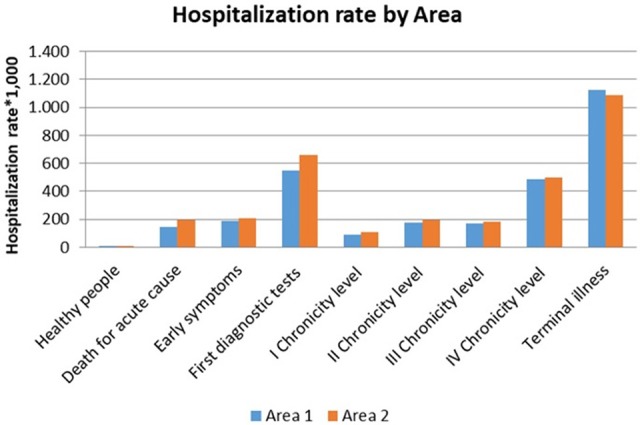
Hospitalization rate per 1.000 (ordinary hospitalization).

Those patients belonging to the “First diagnostic tests” level showed a hospitalization rate greater than 500.0 per 1.000 for both genders and areas (Figure 5).

The hospitalization rates of chronic patients varied from 90.0 to 425.0 per 1.000.

The “Early symptoms” level and the second chronicity level showed comparable hospitalization rates.

Overall hospitalization demand (Tables 1, 2), including ordinary hospitalization, day hospital and day surgery, varied among the complexity levels in both areas. Chronic patients from the fourth level expressed the highest value, followed by those from the first chronicity level and patients in the terminal phase of life.

**Table 1:** Overall hospitalization demand Area 1.

	**Area 1**				
	**(%) of hospitalization demand**	**(%) surgical demand**	**(%) of surgical unplanned hospitalizations**	**(%) medical demand**	**(%) of medical unplanned hospitalizations**
Healthy people	6.0	29.5	74.0	70.5	95.6
Non-chronic cause death	0.2	30.0	66.7	70.0	100.0
Early symptoms	10.8	75.9	27.6	24.1	64.1
First diagnostic tests	1.8	63.6	33.8	36.4	71.8
I Chronicity level	18.7	67.4	30.4	32.6	72.9
II Chronicity level	8.4	45.5	39.9	54.5	74.2
III Chronicity level	9.3	51.4	41.1	48.6	61.9
IV Chronicity level	28.9	29.8	35.8	70.2	57.6
Terminal illness	15.9	14.4	81.3	85.6	79.3
**Total**	**100.0**	**43.3**	**37.6**	**56.7**	**78.8**

**Table 2:** Overall hospitalization demand Area 2.

	**Area2**				
	**(%) of hospitalization demand**	**(%) Surgical demand**	**(%) of surgical unplanned hospitalizations**	**(%) medical demand**	**(%) of medical unplanned hospitalizations**
Healthy people	3.7	17.1	78.8	82.9	96.9
Non-chronic cause death	0.2	16.7	100.0	83.3	90.0
Early symptoms	10.2	79.5	25.9	20.5	58.7
First diagnostic tests	1.6	70.6	41.7	29.4	56.0
I Chronicity level	21.8	65.0	30.7	35.0	76.5
II Chronicity level	8.2	44.5	32.3	55.5	82.4
III Chronicity level	9.8	44.3	36.1	55.7	76.5
IV Chronicity level	30.5	33.2	36.5	66.8	68.8
Terminal illness	14.0	17.1	69.6	82.9	84.3
**Total**	**100.0**	**44.6**	**34.9**	**55.4**	**56.7**

Surgical demand was the most expressed among patient belonging to the “Early symptoms” level to the first chronicity level in both areas, while medical demand mainly concerned “Healthy people” (women giving birth) and “Death for acute cause.”

Among chronic patients, those from the first and the second level of chronicity and the terminal patients showed the highest rate of unplanned hospitalization in the medical area.

### Distribution of ER Access

Our results show that the distribution of access to the ER followed almost the same trend that the hospitalization rate in both areas.

**Figure 6:**
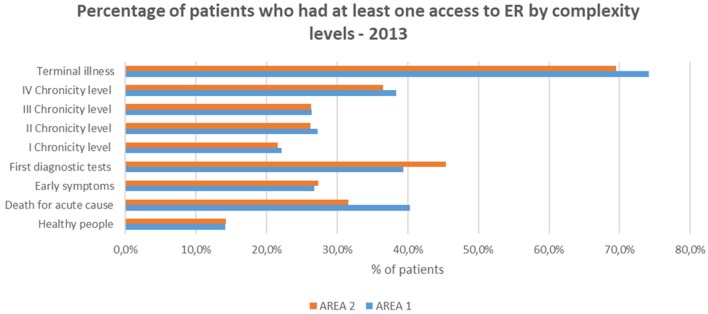
Access to the ER.

The prevalence of patients accessing to the ER was the greatest among those with “Terminal illness,” increasing from the first to the fourth chronicity level (Figure 6).

The subjects belonging to the “First diagnostic tests” level (>39%) showed a rate of access greater than the fourth chronic group (>36%). This could be related to the onset of episodic acute/subacute symptoms which are typical of the early stages of chronicity.

### Home and/or Community Care Service

**Figure 7:**
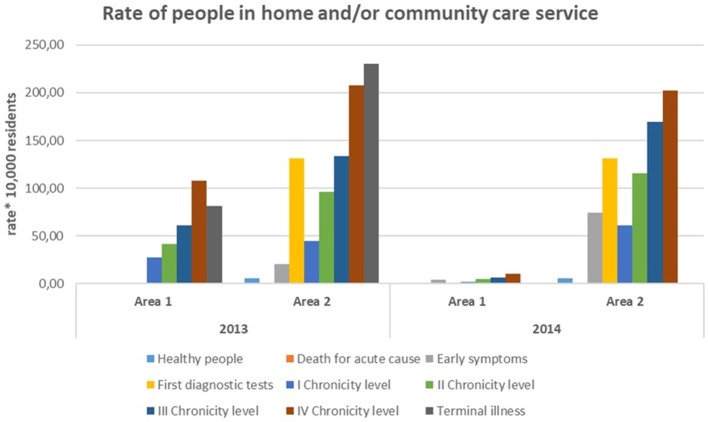
Rate of people in home and/or community care service.

Figure 7 shows that Area 1 and Area 2 had different management of health and social service.

Home/residential management of long-term condition in Area 2 concerned many people in pre-chronic conditions, while in Area1 it was mainly focused on chronic patients.

Area 1 had a lower rate of beds availability (percentage of beds/elderly people), but a greater availability of public economic resources for social care.

### Distribution of Health Spending by Levels of Complexity

**Figure 8:**
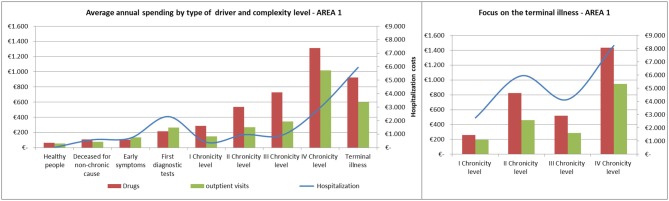
Distribution of health spending by level of complexity.

**Figure 9:**
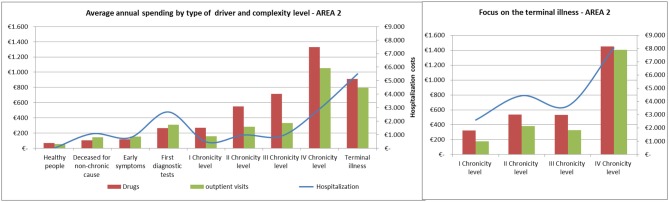
Complexity progression over time.

The average annual spending for drugs, outpatient visits and hospitalization showed comparable trends among the areas (Figures 8, 9).

Spending for drugs increased from the “Healthy people” to the last degree of chronicity. Among the subjects in the “Terminal illness” level, those from the fourth level of chronicity showed the highest values, followed by the patients from the second level of chronicity.

The hospitalization rate among patients in the terminal state followed a similar trend to the pharmaceutical one.

Referring to hospitalization and outpatient visits spending, the subjects belonging to the “First diagnostic tests” showed a peak between 400 and 500 euros/year, on average. Among chronic patients the hospitalization rate did not increased linearly: patients in the second level spent more than those in the third level and subjects belonging to the fourth chronicity level or in terminal state have the highest values.

### Complexity Progression Over Time

Complexity progression over time of each patient derives from the comparison between the subject's complexity level in 2013 and in 2014.

**Figure 10:**
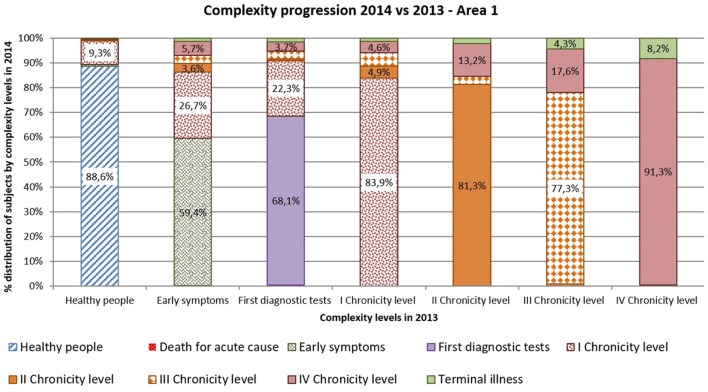
Complexity progression 2014 vs 2013 – Area1.

**Figure 11:**
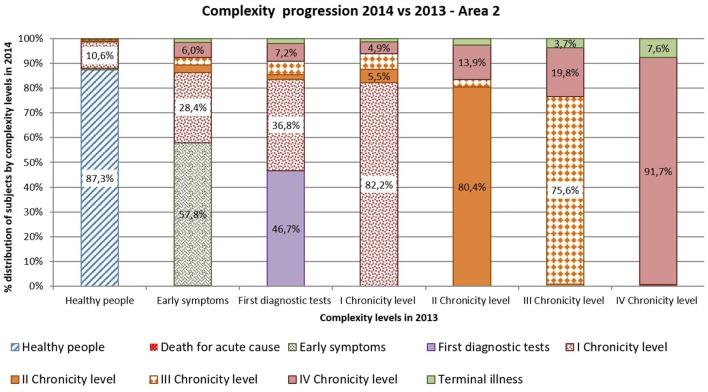
Complexity progression 2014 vs 2013 – Area2.

As shown in Figures 10, 11 more than 9% of healthy subjects in 2013 (first column) progressed to the first chronicity level in the following year (Area1: 11.4%; Area2: 12.7%).

From the “Early symptoms” and “First diagnostic tests” levels (second and third columns), more than 30% of subjects got worsen their health status toward chronicity (Area1: 40.6%; Area2: 42.2%). Those who were already chronic patients in 2013 (from fourth to seventh columns) showed a more stable condition in the following year. More than 83% of the patients maintained the same level of complexity (Area1: 83.9%; Area2: 82.8%).

Comparing areas, the rates of progression were quite similar, excepted for people belonging to the “First diagnostic tests”: subjects from Area 2 showed a faster worsening toward chronicity than those from Area1 (Area1: 31.9%; Area2: 53.3%).

## Discussion

In Italy the 39.1% of population claims to have a chronic disease, the 20.7% has more than one disease and the 80% of the total health expenditure concerns long-term conditions (14).

Further, many studies stated that patients who frequently attended the ER represent subjects who suffer from several comorbidities and require ongoing care (15). Many national and international studies have found that frequent and heavy users of ER are more likely to have chronic illness or “poor health status” and to have higher hospital admission and mortality rates (16–18).

Moreover, in Italy about 9% of dependent elderly population receives long term care in institution, while 42% receives formal care at home. Dependent population receiving informal or no care are still 49% (19).

The long term prevalence among people older than 75 year of age amounts to 83%, comparable to the 2014 ISTAT (Italian National Institute of Statistics) estimate equal to 85.5% (14).

The long-term condition management needs the intervention of various professional figures and the risk that each specialist intervenes in a piecemeal way is real (14).

The traditional disease-focused approach sometimes originates conflicting solutions, with possible diagnostic and therapeutic duplications that increase health expenditure and make it difficult for the patient to participate in the treatment process (14).

Moreover, this approach increases polypharmacy treatment with the real risk of compliance reduction, inappropriate prescriptions, drug interactions and adverse reactions (14).

Conversely, the proactive and longitudinal approach in the long-term condition management is the key for improving patients' quality of life and for cutting down healthcare spending (5).

A proactive approach should be supported by an efficient way to segment population into homogeneous groups, easily identifiable using available standardized data (9).

For this purpose, we developed the IPP algorithm as the preliminary step for identifying groups of population which shares health needs. This could allow a better identification of individual pathways of care for managing chronicity.

Currently our model is limited by the data availability as it uses administrative databases that provides mainly demographics and health/disease history. However, the adopted theoretical model is able to include more data dimensions (lifestyle, genetic profile, environmental exposure, etc.) providing the potential to identify homogeneous groups of subjects with a higher level of precision.

At the moment, a significant aspect of the IPP algorithm is that it is based on data that exists with patient information flow databases which represent not only patients' clinical characteristics, but also the manner in which information is collected and recorded during the healthcare process events, ranging from inpatient admission, inpatient discharge, outpatient visit, emergency department visit and ambulatory surgery (20). The propensity of the variables for clustering based on their association with specific healthcare process events, results in a better derivation of the algorithms, especially for what regards the segmentation (20).

Moreover, the co-morbid profiles were identified by referring to clinical categories rather than to single pathologies because our rationale for the investigation of multi-morbidity is consistent with the hypothesis that the co-occurrence of different classes of disease is more relevant for the management of chronicity, than the number of single diseases belonging to the same clinical category (21).

The application of the IPP segmentation method to different settings of the healthcare (hospital, emergency room, home and/or community care service) and to different geographical areas with similar demographic structure, aimed to validate the model, even though indirectly.

In both areas, as a fact, among the subjects belonging to the chronic levels (from the first to the fourth), the healthcare spending for drugs and outpatient visits grows accordingly to the increase of the complexity, while the hospitalization spending does not follow a linear trend. The second chronic level shows a higher spending than the third one. In particular, the second level, compared to the third one, shows a higher rate of demand in the medical area with a higher rate of acute events (74% of unplanned hospitalization).

The propensity to the hospitalization mainly relates to the fourth level.

This is also the level with (a) the highest spending for drugs and outpatient visits (b) the highest level of hospitalization demand, in particular in the medical area with the 58% of unplanned demand c) the highest rate of people in home/community care service and (d) the most intensive need of multidisciplinary care teams. The fourth level is characterized by the highest rate of co-morbidity due to the combination of those diagnostic categories which required high resources to be managed: cardiovascular and respiratory diseases, neoplasm, and complicated type 2 diabetes.

Our results show that also the “Terminal illness” level, including chronic patients who died during 2013 or 2014, is characterized by high rate of access to ER and high spending for hospitalization. In particular, those patients who died after worsening of the diseases included in the fourth levels, have the highest indexes of spending and use of healthcare services.

According to the Kaiser Permanente Pyramid paradigm, the case management of patients belonging to the fourth and to the terminal levels, regards subjects with multiple long-term conditions, whose complexity makes them high users of healthcare and social services.

In our view some fundamental differences should be highlighted: while the terminal patients should be mainly followed in hospice or within home palliative care service, those patients belonging to the fourth level need more intensive care in the hospital care setting.

On the basis of our results, an average of 8% of patients in both geographical areas and belonging to the fourth level, exacerbates their condition in the following year: this information could allow to better plan more efficient trajectory of treatment in the terminal phase of life.

From a prevention perspective, it is important to consider the patients belonging to the “Early symptoms” and to the “First diagnostic tests” levels. Among those patients who have had at least one or two different hospitalization events per year, more than 30% gets their health status worsen toward chronicity in the following year of observation. They also show a frequent use of ER and a level of annual spending for drugs and outpatient visits which reflects their needs for diagnostic deepening.

A more specific analysis of the outpatient visits or diagnostic needs, could allow to plan integrated day care services, points of care, or other territorial solutions for optimizing the ER use (22) and reducing the waiting times for diagnostic tests.

Moreover, the availability of data about patients' clinical referrals could improve the efficacy of (a) forecasting the individual progression state toward chronicity (b) the strategies for slowing disease progression (proactive approach) and (c) the planning of self-care approaches. This involves education about health condition/s, about strategies to manage changes in own health and about the importance of compliance to drug prescription, in accordance with the Kaiser Permanente Pyramid paradigm.

In particular, the IPP model, providing an integrated overview of the overall condition of the patient, could also give useful indication for the General Medicine.

In Italy, Health Authorities could implement an automatic system which, applying the IPP algorithm to the national administrative databases, support (a) the General Practitioners (GPs) toward a proactive approach to the complexity of their patients and (b) the secondary and tertiary care toward a more comprehensive evaluation of the health history of each patient.

Moreover, the IPP approach could suggest a more efficient allocation of resources to each GP or care services, referring to a system of budgeting rules, identified also taking into account the complexity level of patients attending each clinician or care service.

In conclusion, we propose the IPP model as an innovative method for setting up integrated and multidisciplinary care planning, focusing on the specific profile of individual patients. Additionally, the IPP model proposes a longitudinal approach throughout all the process of disease, from the preventive phase to the terminal one. This can be a critical component of broad population-focused surveillance program in chronicity and it is not reliant on the Electronic Health Records (HER) only, to accomplish its goals.

Furthermore, the IPP approach could be an efficient way for supporting the identification and negotiation of output targets with defined budgets.

However, further validation of the IPP algorithm is expected by extending the dataset capacity to include (a) larger population in order to explore heterogeneity among different geographic settings due to normative aspects (b) longer time of observation for better characterizing complexity progression over time and (c) further data sources availability.

Moreover, we expect a direct form of validation, performing a comparison with the General Practioners' records, referring in particular to those people in the early stage of disease, affected by non-acute health impairment.

Finally, despite the necessity of further validation, we believe the IPP model can be an efficient methodology support for (a) improving performances at the patient's individual level (b) allowing standardized comparison among different geographical areas (c) supporting large population-focused surveillance programs and (d) providing knowledge to identify and fill the gaps in public health policies.

## Ethics Statement

Health Administrative database: the patient's medical record includes written consent given upon admission or before an outpatient visit. Written consent follows the criteria established in the Arts. Thirteen and twenty-second of the Italian Legislative Decree 196/2003 on privacy. All written consent also covers statistical processing of the data for research purposes. In this case data are anonymous and the patient cannot be identified in any way. The linkage procedure among the databases was performed using an anonymized code, created for each patient by the Local Administrative Authority, which is the health provider legally authorized for the processing of sensitive data. According to the Article 2 of Additional Protocol to the Convention on Human Rights and Biomedicine concerning Biomedical Research (http://conventions.coe.int/Treaty/EN/Reports/Html/195.htm), ethics committee approval was not requested because this study involves a secondary analysis of data, processed for the monitoring of the healthcare system.

## Author Contributions

MF provided substantial contributions to the epidemiological conception and design of the work, assisted with acquisition of data and algorithm identification, completed all analysis, led interpretation of data, drafted the work, and revised it critically. SP provided relevant technical contributions to the design of the work, assisted with acquisition of data and algorithm identification, led interpretation of data, and assisted in drafting and revising the work. AC, MC, and SN provided substantial contribution to the design of the work and participated to the final approval of the version to be published. SM assisted in designing the work, drafting the paper, and revising it critically. SM also provided the final approval of the version to be published.

### Conflict of Interest Statement

The authors declare that the research was conducted in the absence of any commercial or financial relationships that could be construed as a potential conflict of interest.
